# The association between intravenous fluid resuscitation and mortality in older emergency department patients with suspected infection

**DOI:** 10.1186/s12245-018-0219-2

**Published:** 2019-01-05

**Authors:** Sin Y. Ko, Laura M. Esteve Cuevas, Merel Willeboer, Annemieke Ansems, Laura C. Blomaard, Jacinta A. Lucke, Simon P. Mooijaart, Bas de Groot

**Affiliations:** 1 Department of Emergency Medicine, Leiden University Medical Center, Albinusdreef 2, 2300 RC Leiden, the Netherlands; 2 Department of Emergency Medicine, Albert Schweitzer Ziekenhuis, Albert Schweitzerplaats 25, 3318 AT Dordrecht, the Netherlands; 3 Department of Gerontology and Geriatrics, Leiden University Medical Center, Albinusdreef 2, 2300 RC Leiden, the Netherlands; 4 Institute for Evidence-based Medicine in Old Age | IEMO, Albinusdreef 2, 2300 RC Leiden, the Netherlands

**Keywords:** Emergency medicine, Geriatrics, Sepsis, Infectious diseases, Fluid resuscitation, Systolic blood pressure

## Abstract

**Objective:**

Recent studies suggest that hypotension thresholds in current guidelines might be too low for older patients due to arterial stiffening, possibly leading to insufficient fluid resuscitation**.** We compared intravenous (IV) fluid volumes that older (≥ 70 years) and younger (< 70 years) patients with suspected infection with similar initial systolic blood pressure (SBP) received in the emergency department (ED) and investigated whether this was associated with in-hospital mortality in older patients.

**Methods:**

This was an observational multicenter study using an existing database in which consecutive ED patients hospitalized with suspected infection were prospectively included. We first compared the fluid volumes older and younger ED patients received per initial SBP category. Patients were then stratified into two SBP categories (≤ or > 120 mmHg; 120 has been suggested to be a better threshold) and thereafter into three fluid volume categories: 0–1 L, 1–2 L, or > 2 L. In each SBP and fluid category, case-mix-adjusted in-hospital mortality was compared between older and younger patients, using multivariable logistic regression analysis.

**Results:**

The included 981 (37%) older and 1678 (63%) younger ED patients received similar IV fluid volumes per initial SBP category. Older patients with an initial SBP > 120 mmHg had a higher adjusted OR of 2.06 (95% CI 1.02–4.16), in the 0–1 L category, while this association was not found in the higher fluid categories of 1–2 L or > 2 L. In the SBP ≤ 120 mmHg category, this association was also absent.

**Conclusion:**

This hypothesis-generating study suggests that older patients with suspected infection may need higher fluid volumes than younger patients, when having a seemingly normal initial SBP.

**Electronic supplementary material:**

The online version of this article (10.1186/s12245-018-0219-2) contains supplementary material, which is available to authorized users.

## Introduction

Fluid resuscitation is an important aspect of sepsis treatment [1–4]. However, the correct timing and the appropriate volumes for resuscitation in the emergency department (ED) is still an active area of debate [5]. Previous studies have primarily focused on intensive care (ICU) patients with severe sepsis and septic shock. Research about fluid resuscitation in the ED, where most patients are in the early stages of sepsis, i.e., before onset of acute organ failure, are scarce. This is especially a problem for older patients as they need higher systolic blood pressures for adequate perfusion due to arterial stiffening [6]. Moreover, because of their blunted heart rate response, their cardiac output mainly depends on cardiac filling pressures with adequate preload [7]. Older patients may therefore require different targets for fluid resuscitation [8]. In clinical practice however, clinicians often withhold a large amount of fluids in older patients because of a fear of overloading the heart [9, 10]. Older and younger patients receive on average similar amounts of fluids [11], despite the fact that age is a risk factor for progression into septic shock [12]. In addition, in most sepsis guidelines, the threshold for hypotension is typically set at systolic blood pressure (SBP) < 90–100 mmHg [13], while older patients are probably already in shock with higher SBPs. Recently, we have shown that in older ED patients with suspected infection, SBP < 140 mmHg is linearly associated with a higher mortality [14]. The same has been suggested in older patients with trauma or surgical sepsis [15, 16]. Therefore, older patients may receive insufficient fluid volumes for adequate perfusion, which could affect outcome.

### Study aim

The aim of this hypothesis-generating study was therefore twofold: Firstly, to investigate how much fluids older ED patients with suspected infection receive compared to younger patients with similar initial SBP. Secondly, to investigate the association between fluid volumes administered in the ED and case-mix-adjusted in-hospital mortality in older compared to younger patients with suspected infection.

## Methods

### Study design and setting

This is an observational multicenter study on an existing database of prospectively collected data as part of an ongoing quality improvement program in three Dutch EDs, which has been described in detail previously (online supplementary file 1) [17]. In the Leiden University Medical Centre (LUMC), data were collected from 1 April 2011 to 1 February 2016, in the Rijnstate Hospital from 1 March 2012 to 1 April 2013, and in the Albert Schweitzer Hospital (ASZ) from 1 September 2015 to 1 December 2015.

For the first aim of the study, we divided the patients into two age groups: < 70 years and ≥ 70 years. We compared how much fluids were administered per initial SBP categories in both groups.

**Fig. 1:**
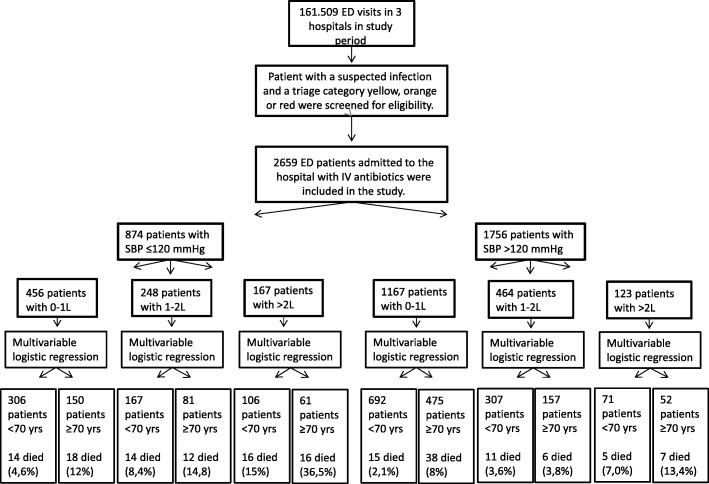
Patient flow through study and stratification design for multivariable logistic regression analysis. Patients were first stratified in two groups according to initial SBP. A threshold of 120 mmHg is chosen to investigate whether a higher SBP threshold is needed for older patients. Patients were subsequently stratified into three fluid categories. Multivariable logistic regression analysis was performed per group to compare in-hospital mortality in older patients to younger patients, adjusting for confounders. Here, we show which groups were compared in the multivariable regression analysis of Table 2. The results of the multivariable analysis are shown in Table 2. Abbreviations: ED = emergency department, IV = intravenous, SBP = systolic blood pressure, L = liter, yrs = years

For the second aim of the study, we first had to stratify patients according to their initial SBP, because low SBP is an important indicator to initiate fluid resuscitation. We stratified patients into a group with a low SBP (≤ 120 mmHg) or a “seemingly normal” SBP (> 120 mmHg), based on the study by Oyentunji et al. [16], in which it has been suggested that 120 mmHg is a hypotension threshold for older patients. Secondly, fluid volume itself is a measure of disease severity not captured in initial disease severity scores because it partially reflects response to ED treatment. In multivariable regression models, fluid volume administered in the ED has been found to be an independent predictor of mortality [18]. Hence, patients who received high fluid volumes could not simply be compared to those who received low fluid volumes. It has also been suggested that older and younger patients receive similar amount of fluids [11]. Therefore, to investigate whether the current fluid resuscitation strategy, in which older and younger patients receive similar amount of fluids, is associated with a higher mortality in older patients, we had to divide patients into three fluid volume groups: 0–1 L, 1–2 L and > 2 L. Older patients were compared to younger patients in the same fluid category and same initial SBP category. The stratification is shown in Fig. 1.

#### Selection of participants

Consecutive patients ≥ 17 years and urgent triage categories who were hospitalized with a suspected infection after receiving intravenous antibiotics in the ED were included. There were no exclusion criteria.

The study was approved by the medical ethics committee of the LUMC.

### Methods and measurements

Demographics, comorbidities, laboratory values, vital signs, treatment administered (including antibiotics, intravenous fluids, and oxygen), disposition from the ED, and outcomes were collected as described previously [17].

SBP was measured non-invasively with the MP52 monitor (Philips, Eindhoven, The Netherlands) in LUMC, with the M8007A Intellivue (Philips, Eindhoven, The Netherlands) in ASZ, and with the Infinity C700/M540 (Dräger medical systems, Telford, USA) in Rijnstate Hospital. Initial SBP was measured within half an hour after ED registration and divided in four categories: ≤ 100, 101–120, 121–140, and ≥ 140 mmHg, based on the threshold for hypotension in the quick Sequential Organ Failure Assessment Score (qSOFA) and the mean SBP in the general Dutch population of ≥ 70 years [13, 19].

The Predisposition, Infection, Response and Organ failure (PIRO) score was used as a measure of disease severity, taking into account demographics, comorbidities, and acute physiology parameters [20, 21].

Admission to a medium or intensive care unit (MICU) was used as a reflection of response to ED treatment and disease severity, not captured in the initial PIRO score.

#### Fluid administration

All types of fluids, i.e., colloids and crystalloids (mostly NaCl 0.9%), were used. If patients had arrived by ambulance, the amount of fluids in the ambulance was taken into account and added to the amount given during the total ED stay. If a registration form was missing, it was assumed that no fluid was given. Fluid volumes administered after ED departure were not registered.

The primary outcome measure was in-hospital mortality.

### Data analysis

#### Sample size

The second aim of the study required the largest sample size and was therefore used to calculate the necessary number of events. Approximately 5–9 events per variable have been shown to be acceptable in association studies [22]. We corrected for 5 confounders; hence, we needed approximately 25 events per group. Because the second part of the study was intended as hypothesis generating, smaller number of events was considered to be acceptable as long as the regression model did not give unacceptably high 95% confidence intervals (CI).

#### Descriptive statistics

Data were presented as mean (standard deviation (SD)) when normally distributed. Skewed data were presented as median (interquartile range (IQR)). Categorical data were presented as number (%).

#### Main analysis

For the first aim of the study, the association between the initial systolic blood pressure and the amount of fluid administered in the ED was assessed through a line graph with 95% CI bars, comparing older and younger patients.

For the second aim of the study, patients were divided into two initial SBP categories: ≤ 120 and > 120 mmHg. The patients in the two SBP categories were thereafter split into three different fluid categories. An association model [23], as opposed to a prediction model, was developed per fluid category in order to explain the relation between age and outcome in patients, who received similar fluid volumes and presented in the same initial SBP category. Based on previous studies [17, 18], the following confounders were entered into the model: type of hospital (urban vs academic), oxygen administration, “Do Not Resuscitate (DNR) code”, the Predisposition and Infection (PI) score and Response and Organ Failure (RO) score [20], and MICU admission. The PI score reflects the potentially non-modifiable aspects, taking age, comorbidities, and type of infection into account, while the RO score represents the modifiable aspects, based on acute physiology parameters and extent of organ failure [21, 24]. These variables all met the criteria to be a potential confounder as they are associated with the primary determinant and the outcome [25]. To investigate if the primary association of interest could be corrected for less confounders (because of the limited number of event per variable), we first entered each of the predefined potential confounders into the model with age and outcome. The variable resulting in the largest change in the regression coefficient of the association age and outcome was then added to the model, which subsequently became the new starting model. This procedure was repeated until the addition of a new variable changed the regression coefficient of the primary association of interest with less than 10%, which was considered irrelevant. Multivariable logistic regressions were performed separately for the three fluid categories, each time comparing the older group to the younger group, with the younger group as reference. This data analysis design (Fig. 1) will answer the question of whether older and younger patients receiving similar fluid volumes at the same initial SBP affects in-hospital mortality in older patients.

Odds ratios (OR) are reported with 95% confidence intervals (95% CI). An *α* of 0.05 was used to distinguish statistically significant results. Data were processed using SPSS (SPSS, version 23.0, IBM, New York, USA).

#### Sensitivity analyses

We performed several sensitivity analyses: First, to investigate the impact of the number of variables in the final model on effect size, we did a sensitivity analysis in which we excluded the variables with the smallest impact on the regression coefficient (see Additional file 1). Secondly, chronic heart disease was also added into the association model building process to see if it had an influence on the association age and outcome (see Additional file 2).

## Results

### Patient characteristics

Table 1 shows that 1678 (63%) of the 2659 included patients were < 70 years and 981 (37%) were ≥ 70 years. Older patients had a lower heart rate, higher SBP, and higher PIRO score.

**Table 1:** Patients’ characteristics

	Total cohort *N* = 2659	< 70 years *N* = 1678	≥ 70 years *N* = 981	*p* value
Patient demographics				
Age, mean (SD)	62.1 (17.1)	52.5 (13.6)	78.7 (6.3)	0,000
Sex, *n* (%)				
Female	1143 (43.0)	766 (45.6)	377 (38.4)	0.000
Male	1516 (57.0)	912 (54.4)	604 (61.6)	
Comorbidities, *n* (%)				
COPD (2)	416 (15.6)	185 (11.0)	231 (23.5)	0.000
Heart disease (1)	432 (16.1)	156 (9.3)	276 (28.1)	0.000
Kidney disease (1)	494 (18.5)	282 (16.8)	212 (21.6)	0.002
Liver disease	125 (4.7)	102 (6.1)	23 (2.3)	0.000
Malignancy	280 (10.5)	173 (10.3)	107 (10.9)	0.628
Metastatic malignancy	37 (13.9)	256 (15.3)	117 (11.9)	0.017
Triage category (2), *n* (%)				0.174
Blue	2 (0.1)	0	2 (0.2)	
Green	7 (0.3)	5 (0.3)	2 (0.2)	
Yellow	1233 (46.4)	780 (46.5)	453 (46.2)	
Orange	1365 (51.3)	866 (51.6)	499 (50.9)	
Red	50 (1.9)	26 (1.5)	24 (2.4)	
Suspected site of infection, *n* (%)				
Lungs	1319 (49.3%)	736 (43.9)	583 (59.4)	0.000
Urogenital tract	797 (29.8)	453 (27.0)	344 (35.1)	0.000
Abdomen	459 (17.2)	305 (18.2)	154 (15.7)	0.306
Skin	245 (9.2)	165 (9.8)	80 (8.2)	0.239
CNS	68 (2.5)	47 (2.8)	21 (2.1)	0.369
Other	432 (16.1)	323 (19.3)	109 (11.1)	0.000
Vital signs*				
Respiratory rate, mean (SD) (590)	24 (7)	23 (7)	26 (7)	0.158
Heart rate, mean (SD) (46)	108 (20)	111 (19)	103 (20)	0.112
MAP, median (IQR) (247)	94 (83–105)	94 (84–105)	94 (84–105)	0.528
Systolic BP, median (IQR) (29)	130 (115–149)	129 (115–145)	135 (115–154)	0.000
Disease severity, median (IQR)				
PIRO score	10 (6–14)	8 (4–12)	13 (9–16)	0.000
Lactate (mmol/L) (286)	1.8 (1.4–0.6)	1.80 (1.4–2.5)	1.90 (1.4–2.7)	0.023
Total amount of fluids received (L) (6)	1.00 (0.5–1.5)	1.00 (0.5–1.5)	1.05 (0.5–1.5)	0.128
Supplemental oxygen (L/min)** (78)	2 (0–5)	2 (0–4)	3 (1–5)	0.000
Mortality, *n* (%)	172 (6.5)	75 (4.5)	97 (9.9)	0.000

Data are presented as mean (SD), when normally distributed, or as median (IQR) if rightly skewed. Categorical data were presented as number (%). The two age cohorts were compared using the unpaired *t* test, Kruskal-Wallis test, or chi-square test depending on the variable. The significance was presented by the *p* value. The number between brackets indicate the amount of missing variables

*COPD* chronic obstructive pulmonary disease, *CNS* central nervous system, *MAP* mean arterial pressure, *BP* blood pressure, *PIRO* Predisposition, Infection, Response, Organ failure, *SD* standard deviation, *IQR* interquartile range

*Vital signs registered before treatment at emergency department

**The maximum amount of oxygen that was administered at the emergency department

### Main results

**Fig. 2:**
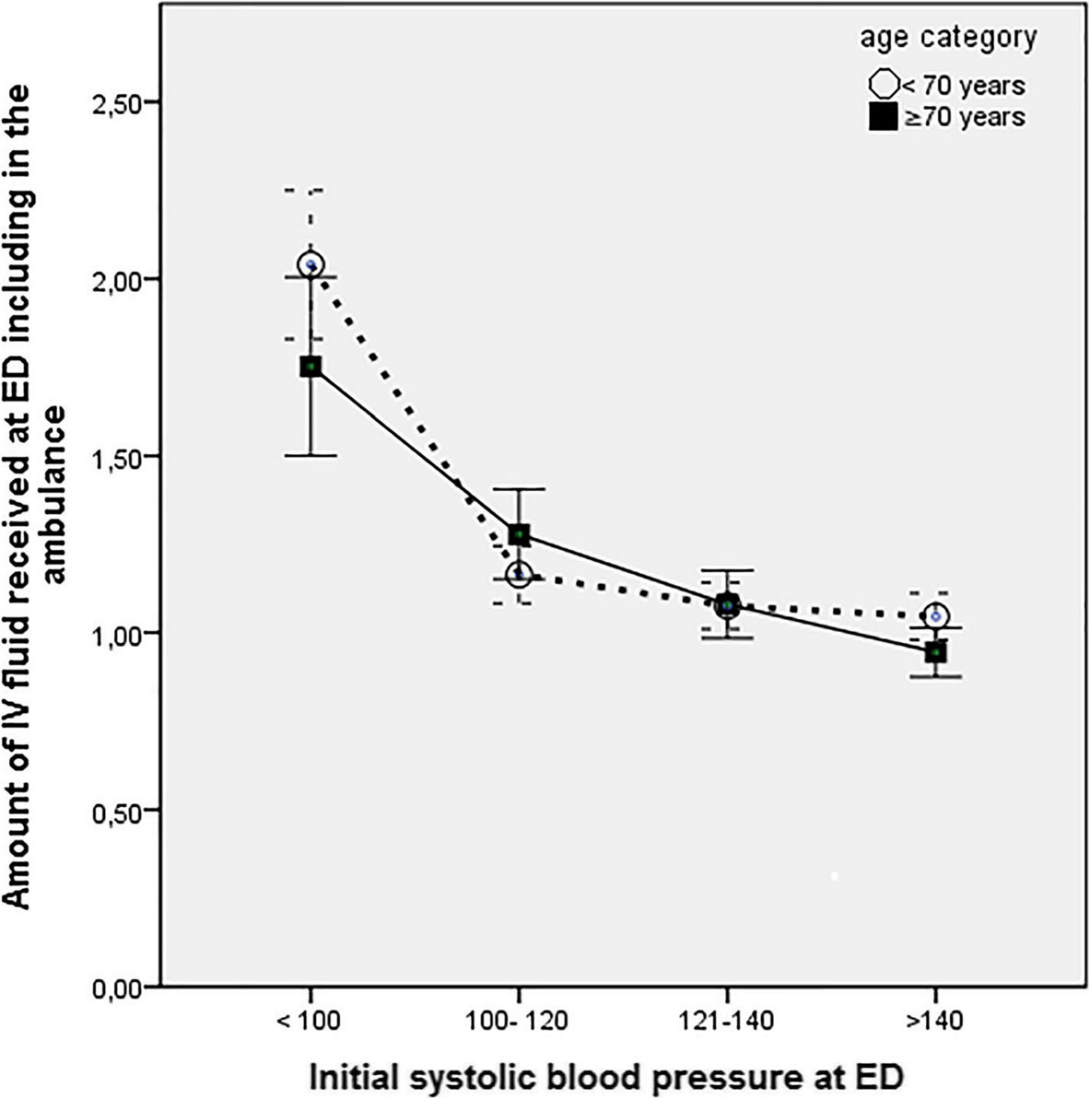
Administered fluid volumes per initial systolic blood pressure category. The mean amount of fluid administered at the emergency department is the same for younger and older patients in every initial systolic blood pressure (mmHg) category. The dotted line with the open circles represents patients < 70 years. The solid line with closed squares represents patients ≥ 70 years. Abbreviations: IV = intravenous, ED = emergency department

In Fig. 2, it is shown that older and younger patients receive similar fluid volumes in all initial SBP categories. Administered fluid volumes increased with decreasing SBP category. We did not measure ED lengths of stay in this study but the median (IQR) LOS in our ED is 156 (98–225) min [26].

Table 2 shows that older patients in the fluid volume categories 0–1 L and 1–2 L needed more supplemental oxygen and had a higher RO score.

**Table 2:** Characteristics per fluid category divided by age category

	Intravenous fluids received in ED and ambulance (L)					
	< 70 years 0–1	≥ 70 years 0–1	< 70 years 1–2	≥ 70 years 1–2	< 70 years > 2	≥ 70 years > 2
Patients (8), *n*	1021	627	476	238	177	114
Absolute mortality (14), *n* (%)	31 (3.0)	56 (8.9)	25 (5.3)	18 (7.6)	21 (11.9)	23 (20.2)
PIRO score, median (IQR)	8 (4–12)	13 (9–15)	8 (4–12)	13 (9–15)	11 (7–14.5)	14 (10–17)
PI score, median (IQR)	4 (2–5)	6 (4–8)	4 (2–5)	6 (4–7)	3 (2–5)	5 (3–7)
RO score (IQR)	3 (2–8)	7 (3–9)	4 (2–8)	8 (4–9)	8 (4–10)	8 (5–11.25)
Supplemental oxygen (L/min) (23), median (IQR)	0 (0–3)	3 (1–5)	2 (0–5)	4 (2–7.5)	5 (2–12.5)	5 (3–15)
Do Not Resuscitate status (3), *n* (%)	127 (12.5)	266 (42.4)	53 (11.1)	103 (43.3)	29 (16.4)	46 (40.4)

The variables and outcomes included into the association model per fluid category and per age category

The number between brackets indicate the amount of missing variables

*ED* emergency department, *n* number, *IQR* interquartile range

The association between age and in-hospital mortality in different fluid volume categories, adjusted for potential confounders, divided into initial SBP categories, is shown in Table 3. The younger age group was set as a reference, to which the older patients were compared. In the ≤ 120 mmHg category, the ORs were approximately 1 in all fluid categories (*P* > 0.05). In the SBP > 120 mmHg category on the other hand, older patients had an adjusted OR of 2.06 (1.02–4.16) for in-hospital mortality, when they received 0–1 L of fluids. Forty-eight percent of the older patients fell into this category. Older patients receiving 1–2 L had an adjusted OR of 0.28 (0.07–1.04). The OR in > 2 L was not significant.

**Table 3:** Adjusted odds ratios for in-hospital mortality per SBP category

IV fluids received at ED including in the ambulance (L)	OR (95% CI)	*p* value
SBP ≤ 120 mmHg		
0–1 L	1.18 (0.47–2.97)	0.726
1–2 L	0.78 (0.26–2.15)	0.644
> L	1.08 (0.43–2.70)	0.868
SBP > 120 mmHg		
0–1 L	2.06 (1.02–4.16)	0.045
1–2 L	0.28 (0.07–1.04)	0.057
> 2 L	2.35 (0.59–9.45)	0.228

Association model was made for age category and in-hospital mortality, per three fluid categories, and split per blood pressure category. Adjusted for Do Not Resuscitate status, urban or academic hospital, PI score, RO score, and supplemental oxygen. Figure 1 shows the stratification into the different groups

*IV* intravenous, *ED* emergency department, *OR* odds ratios, *CI* confidence interval, *SBP* initial systolic blood pressure, *PI* Predisposition and Infection, *RO* Response and Organ failure

### Sensitivity analyses

Fewer variables in the model had no impact on the effect size of the primary association (Additional file 1). The comorbidity “chronic heart disease” did not influence the association between age and outcome in all fluid categories (Additional file 2).

## Discussion

In conclusion, despite twice as high organ dysfunction in the lower fluid volume categories, older ED patients hospitalized with a suspected infection receive similar fluid volumes per initial SBP category compared to younger patients. In the group receiving low fluid volumes, case-mix-adjusted mortality was twice as high in older compared to younger patients with a “seemingly normal” SBP of > 120 mmHg suggesting that older patients may need higher fluid volumes during ED resuscitation.

To the best of our knowledge, no other investigations have studied the impact of different fluid volumes in older patients with early stages of sepsis, while in older patients with severe sepsis or septic shock aggressive fluid resuscitation improves survival [27, 28].

The initial RO score, reflecting potentially reversible aspects of disease severity, was more than twice as high in older compared to younger ED patients in lower fluid categories. We hypothesize that physicians in the ED do not administer higher fluid volumes during ED resuscitation because they do not timely recognize initial disease severity in older patients with a suspected infection, possibly because the initial seemingly normal SBP of higher than 120 mmHg is not interpreted as hypotension. In our hypothesis-generating study, it is suggested that fluid volumes lower than 1 L are associated with higher in-hospital mortality in older patients with initial SBP > 120 mmHg. The absence of an association with case-mix adjusted mortality in older patients with higher fluid volumes could be explained by the higher preload resulting in higher stroke volumes due to the Frank-Starling mechanism. This may be even more important for older patients with chronic heart failure with preserved ejection fraction as long as fluids are cautiously titrated to effect due to small margins between hypovolemia and fluid overload.

Older patients who received 1–2 L tended to have reduced odds for in-hospital mortality compared to younger patients. However, the small number of events in this group, resulting in a wide 95% CI and the inconsistency in significance of the OR in the sensitivity analyses, indicates that further research is needed to investigate this potential beneficial effect of larger fluid volumes in older patients. In current guidelines, no difference exists between older and younger adults with respect to fluid resuscitation [1], even though increasing evidence shows that older patients often have atypical disease presentation and may benefit from a different approach [8]. Our findings suggest that further research is needed to assess what fluid resuscitation strategy is best for older patients with suspected infection.

Our study has its strengths, like the prospective data collection in multiple hospitals. For the first aim of the study, the investigation was conducted in a large cohort. However, it also has several limitations. This is an observational study, which complicates the discrimination between cause and effect. The second aim of the study was mainly explorative and meant to be hypothesis generating. To mitigate the effect of disease severity on fluid administration, we had to stratify patients into similar SBP and fluid categories, limiting the power of the second part of the study. It is important to acknowledge the different nature of an association model as opposed to a prediction model [29]. It has been suggested that the rule of thumb of 10 events per variable is not as strict for association models [22]. Because of the explorative nature of the second part of the study, we did not want to ignore potential important associations by using criteria that might be too strict. Nonetheless, future studies need to confirm our findings in larger patient cohorts.

## Conclusions

In conclusion, this study suggests that older patients receive similar amount of fluids as younger patients do during ED resuscitation regardless of initial SBP while older patients were observed to have twice more adjusted odds for case-mix-adjusted in-hospital mortality compared to their younger control patients, when they received < 1 L of fluids, while presenting with an initial SBP > 120 mmHg. Future studies should focus on the effect of more aggressive early fluid resuscitation in older ED patients with suspected infection.

## Additional files

Additional file 1: Sensitivity analyses investigating the impact of fewer variables on effect size. Sensitivity analyses were performed with fewer variables in the regression model to assess the impact of number of variables on effect size. Fewer variables had no impact on the association of interest. (DOCX 15 kb)

Additional file 2: Sensitivity analyses investigating the impact of incorporation of the variable “chronic heart disease” on effect size. Sensitivity analyses were performed by adding “chronic heart disease” into the association model. Chronic heart disease had too little effect on the regression coefficient to be included into the model and so did not significantly affect the association of interest. (DOCX 13 kb)

## Abbreviations

ASZ: Albert Schweiter Hospital
CI: Confidence interval
DNR: Do Not Resuscitate
ED: Emergency department
ICU: Intensive care
IQR: Interquartile range
LUMC: Leiden University Medical Centre
MICU: Medium or intensive care unit
OR: Odds ratio
PI: Predisposition and Infection
PIRO: Predisposition, Infection, Response and Organ failure
qSOFA: Quick Sequential Organ Failure Assessment Score
RO: Response and Organ Failure
SBP: Systolic blood pressure
SD: Standard deviation

## References

[1] Rhodes A, Evans LE, Alhazzani W (2017). Surviving Sepsis Campaign: International Guidelines for Management of Sepsis and Septic Shock 2016. Crit Care Med.

[2] Rivers E, Nguyen B, Havstad S (2001). Early goal-directed therapy in the treatment of severe sepsis and septic shock. N Engl J Med.

[3] The ARISE investigators and the ANZICS trials group (2014). Goal-directed resuscitation for patients with early septic shock. N Engl J Med.

[4] Mouncey PR, Osborn TM, Power SG (2015). Trial of early, goal-directed resuscitation for septic shock. N Engl J Med.

[5] Harris T, Coats TJ, Elwan MH (2018). Fluid therapy in the emergency department: an expert practice review. Emerg Med J.

[6] Strait JB, Lakatta EG (2012). Aging-associated cardiovascular changes and their relationship to heart failure. Heart Fail Clin.

[7] Nasa P, Juneja D, Singh O (2012). Severe sepsis and septic shock in the elderly: an overview. World J Crit Care Med.

[8] Girard TD, Opal SM, Ely EW (2005). Insights into severe sepsis in older patients: from epidemiology to evidence-based management. Clin Infect Dis.

[9] Liu VX, Morehouse JW, Marelich GP (2016). Multicenter implementation of a treatment bundle for patients with sepsis and intermediate lactate values. Am J Respir Crit Care Med.

[10] Yoshikawa TT, Norman DC (2000). Acute emergencies and critical care of the geriatric patient.

[11] de Groot B, Stolwijk F, Warmerdam M (2017). The most commonly used disease severity scores are inappropriate for risk stratification of older emergency department sepsis patients: an observational multi-centre study. Scand J Trauma Resusc Emerg Med.

[12] Glickman SW, Cairns CB, Otero RM (2010). Disease progression in hemodynamically stable patients presenting to the emergency department with sepsis. Acad Emerg Med.

[13] Singer M, Deutschman CS, Seymour CW (2016). The third international consensus definitions for sepsis and septic shock (sepsis-3). JAMA.

[14] Warmerdam M, Baris L, van Liebergen, et al. The association between systolic blood pressure and in-hospital mortality in older emergency department patients who are hospitalised with a suspected infection. Emerg Med J. 2018;35(10):619–22. 10.1136/emermed-2018-207502 29982193

[15] Clarke DL, Chipps JA, Sartorius B (2016). Mortality rates increase dramatically below a systolic blood pressure of 105-mm Hg in septic surgical patients. Am J Surg.

[16] Oyetunji TA, Chang DC, Crompton JG (2011). Redefining hypotension in the elderly: normotension is not reassuring. Arch Surg.

[17] De Groot B, Struyk B, Najafi R (2017). Inclusion of emergency department patients in early stages of sepsis in a quality improvement programme has the potential to improve survival: a prospective dual-centre study. Emerg Med J.

[18] de Groot B, Ansems A, Gerling DH (2015). The association between time to antibiotics and relevant clinical outcomes in emergency department patients with various stages of sepsis: a prospective multi-center study. Crit Care.

[19] Volksgezondheidenzorg.info. Bloeddruk, Cijfers & Context, Huidige situatie. https://www.volksgezondheidenzorg.info/onderwerp/bloeddruk/cijfers-context/huidige-situatie#!node-systolische-bloeddruk-naar-leeftijd. Accessed 18 Nov 2017.

[20] de Groot B, Lameijer J, de Deckere ER (2014). The prognostic performance of the predisposition, infection, response and organ failure (PIRO) classification in high-risk and low-risk emergency department sepsis populations: comparison with clinical judgement and sepsis category. Emerg Med J.

[21] Howell MD, Talmor D, Schuetz P (2011). Proof of principle: the predisposition, infection, response, organ failure sepsis staging system. Crit Care Med.

[22] Vittinghoff E, McCulloch CE (2007). Relaxing the rule of ten events per variable in logistic and cox regression. Am J Epidemiol.

[23] Twisk J (2006). Applied multilevel analysis.

[24] Levy MM, Fink MP, Marshall JC (2003). 2001 SCCM/ESICM/ACCP/ATS/SIS International Sepsis Definitions Conference. Intensive Care Med.

[25] Lederer DJ, Bell SC, Branson RD (2009). Control of confounding and reporting of results in causal inference studies: guidance for authors from editors of respiratory, sleep, and critical care journals. Ann Thorac Med.

[26] van der Veen D, Remeijer C, Fogteloo AJ (2018). Independent determinants of prolonged emergency department length of stay in a tertiary care centre: a prospective cohort study. Scand J Trauma Resusc Emerg Med.

[27] El Solh AA, Akinussi ME, Alsawalha LN (2008). Outcome of septic shock in older adults after implementation of the sepsis “bundle”. J Am Geriatr Soc.

[28] Heppner HJ, Singler K, Kwetkat A (2012). Do clinical guidelines improve management of sepsis in critically ill elderly patients? A before-and-after study of the implementation of a sepsis protocol. Wien Klin Wochenschr.

[29] Shmueli G (2010). To explain or to predict?. Stat Sci.

