# Supplementary material for: The association between intravenous fluid resuscitation and mortality in older emergency department patients with suspected infection
Source: Int J Emerg Med. 2019 Jan 5;12:1. doi: 10.1186/s12245-018-0219-2 (PMC6326108; doi:10.1186/s12245-018-0219-2)
Supplement: Supplementary file 1 — Sensitivity analyses investigating the impact of fewer variables on effect size. Sensitivity analyses were performed with fewer variables in the regression model to assess the impact of number of variables on effect size. Fewer variables had no impact on the association of interest. (DOCX 15 kb) [file 12245_2018_219_MOESM1_ESM.docx]

**Additional file 1. Sensitivity analyses with fewer variables**

Sensitivity analyses were performed by fewer variables to assess the impact of number of variables on effect size.

| **Table A1. Adjusted odds ratios for in-hospital mortality per SBP category** | | | |
| --- | --- | --- | --- |
| IV fluids received at ED including in the ambulance (L) | **OR** | **95% CI** | **p-value** |
| **SBP ≤ 120 mmhg** |  |  |  |
| 0-1 L | 1.040 | 0.428 – 2.529 | 0.930 |
| 1-2 L | 0.633 | 0.229 – 1.750 | 0.378 |
| >2 L | 1.349 | 0.565 – 3.223 | 0.501 |
| **SBP > 120 mmhg** |  |  |  |
| 0-1 L | 1.873 | 0.941 – 3.728 | 0.074 |
| 1-2 L | 0.245 | 0.067 – 0.893 | 0.033 |
| >2 L | 1.724 | 0.468 – 6.360 | 0.413 |
| Adjusted for DNR-status, PI-score, RO-score  Abbreviations: SBP = systolic blood pressure, IV= intravenous, ED = emergency department, L= liter, OR= odds ratio, CI = confidence interval   \| **Table A2. Adjusted odds ratios for in-hospital mortality per SBP category** \| \| \| \| \| --- \| --- \| --- \| --- \| \| IV fluids received at ED including in the ambulance (L) \| **OR** \| **95% CI** \| **p-value** \| \| **SBP ≤ 120 mmhg** \|  \|  \|  \| \| 0-1 L \| 1.748 \| 0.740 – 4.128 \| 0.203 \| \| 1-2 L \| 0.901 \| 0.336 – 2.419 \| 0.836 \| \| >2 L \| 1.100 \| 0.447 – 2.708 \| 0.835 \| \| **SBP > 120 mmhg** \|  \|  \|  \| \| 0-1 L \| 2.764 \| 1.401 – 5.453 \| 0.003 \| \| 1-2 L \| 0.264 \| 0.074 – 0.940 \| 0.040 \| \| >2 L \| 1.941 \| 0.518 – 7.269 \| 0.325 \| \| Adjusted for DNR-status, PI-score, supplemental oxygen  Abbreviations: SBP = systolic blood pressure, IV= intravenous, ED = emergency department, L= liter, OR= odds ratio, CI = confidence interval \| \| \| \| | | | |

| **Table A3. Adjusted odds ratios for in-hospital mortality per SBP category** | | | |
| --- | --- | --- | --- |
| IV fluids received at ED including in the ambulance (L) | **OR** | **95% CI** | **p-value** |
| **SBP ≤ 120 mmhg** |  |  |  |
| 0-1 L | 1.151 | 0.459 – 2.885 | 0.764 |
| 1-2 L | 0.749 | 0.266 – 2.110 | 0.584 |
| >2 L | 1.091 | 0.439 – 2.713 | 0.851 |
| **SBP > 120 mmhg** |  |  |  |
| 0-1 L | 2.096 | 1.037 – 4.236 | 0.039 |
| 1-2 L | 0.252 | 0.070 – 0.906 | 0.035 |
| >2 L | 1.912 | 0.512 – 7.138 | 0.335 |
| Adjusted for DNR-status, PI-score, RO-score, supplemental oxygen  Abbreviations: SBP = systolic blood pressure, IV= intravenous, ED = emergency department, L= liter, OR= odds ratio, CI = confidence interval | | | |

| **Table A5. Adjusted odds ratios for in-hospital mortality per SBP category** | | | |
| --- | --- | --- | --- |
| IV fluids received at ED including in the ambulance (L) | **OR** | **95% CI** | **p-value** |
| **SBP ≤ 120 mmhg** |  |  |  |
| 0-1 L | 1.013 | 0.413 – 2.487 | 0.977 |
| 1-2 L | 0.671 | 0.897 – 1.316 | 0.394 |
| >2 L | 1.489 | 0.604 – 3.674 | 0.387 |
| **SBP > 120 mmhg** |  |  |  |
| 0-1 L | 2.128 | 1.063 – 4.253 | 0.033 |
| 1-2 L | 0.350 | 0.094 – 1.301 | 0.117 |
| >2 L | 1.994 | 0.525 – 7.569 | 0.311 |
| Adjusted for DNR-status, PI-score, RO-score, ICU or MC admission  Abbreviations: SBP = systolic blood pressure, IV= intravenous, ED = emergency department, L= liter, OR= odds ratio, CI = confidence interval | | | |

| **Table A6. Adjusted odds ratios for in-hospital mortality per SBP category** | | | |
| --- | --- | --- | --- |
| IV fluids received at ED including in the ambulance (L) | **OR** | **95% CI** | **p-value** |
| **SBP ≤ 120 mmhg** |  |  |  |
| 0-1 L | 1.130 | 0.458 – 2.787 | 0.791 |
| 1-2 L | 0.792 | 0.925 – 1.345 | 0.639 |
| >2 L | 1.402 | 0.580 – 3.388 | 0.453 |
| **SBP > 120 mmhg** |  |  |  |
| 0-1 L | 2.341 | 1.063 – 4.253 | 0.016 |
| 1-2 L | 0.358 | 0.098 – 1.315 | 0.122 |
| >2+ L | 2.027 | 0.535 – 7.684 | 0.299 |
| Adjusted for DNR-status, PI-score, ICU or MC admission | | | |

Abbreviations: SBP = systolic blood pressure, IV= intravenous, ED = emergency department, L= liter, OR= odds ratio, CI = confidence interval
