# Supplementary material for: The association between intravenous fluid resuscitation and mortality in older emergency department patients with suspected infection
Source: Int J Emerg Med. 2019 Jan 5;12:1. doi: 10.1186/s12245-018-0219-2 (PMC6326108; doi:10.1186/s12245-018-0219-2)
Supplement: Supplementary file 2 — Sensitivity analyses investigating the impact of incorporation of the variable “chronic heart disease” on effect size. Sensitivity analyses were performed by adding “chronic heart disease” into the association model. Chronic heart disease had too little effect on the regression coefficient to be included into the model and so did not significantly affect the association of interest. (DOCX 13 kb) [file 12245_2018_219_MOESM2_ESM.docx]

**Additional file 2.** Sensitivity analyses with heart diseases
Sensitivity analyses were performed by adding heart disease into the association model building. process.

| **Appendix B1**. Association model for 0-1 L | | |
| --- | --- | --- |
| Variables included in the model | **OR (95% CI)** | **p-value** |
| **SBP ≤ 120 mmHg** |  |  |
| Age category | 1.01 (0.41 – 2.49) | 0.977 |
| PI-SCORE | 1.10 (0.93 – 1.31) | 0.268 |
| DNR-status | 2.61 (1.07 – 6.36) | 0.034 |
| MICU admission | 3.36 (1.20 – 9.46) | 0.022 |
| RO-score | 1.16 (1.03 – 1.30) | 0.014 |
| **SBP > 120 mmHg** |  |  |
| Age category | 2.13 (1.06 – 4.25) | 0.033 |
| PI-SCORE | 1.04 (0.91 – 1.19) | 0.562 |
| DNR-status | 4.16 (2.18 – 7.96) | 0.000 |
| MICU admission | 3.97 (1.63 – 9.64) | 0.002 |
| RO-score | 1.11 (1.01 – 1.22) | 0.027 |
| For a sensitivity analysis, an association model was built taking heart diseases into the consideration. In the group of patients who received 0-1L, heart diseases had too little effect on the regression coefficient to be included into the model and so did not affect the association of concern. Abbreviations OR= odds ratios, CI= confidence interval, SBP= initial systolic blood pressure, L = liter, PI = Predisposition, Infection, RO = Response, Organ failure, DNR= Do Not Resuscitate, MICU= medium care or intensive care | | |

| **Appendix B2**. Association model for 1-2 L | | |
| --- | --- | --- |
| Variables included in the model | **OR (95% CI)** | **p-value** |
| **SBP ≤ 120 mmHg** |  |  |
| Age category | 0.80 (0.30 – 2.12) | 0.797 |
| PI-SCORE | 1.12 (0.92 – 1.35) | 0.256 |
| DNR-status | 8.63 (3.16 – 23.57) | 0.000 |
| MICU admission | 3.21 (1.04 – 9.91) | 0.042 |
| Heart diseases | 0.94 (0.32 – 2.83) | 0.918 |
| **SBP > 120 mmHg** |  |  |
| Age category | 0.42 (0.11 – 1.61) | 0.205 |
| PI-SCORE | 1.38 (1.05 – 1.81) | 0.022 |
| DNR-status | 3.92 (1.12 – 13.70) | 0.033 |
| MICU admission | 15.39 (4.67 – 50.75) | 0.000 |
| Heart diseases | 0.33 (0.39 – 2.84) | 0.331 |
| For a sensitivity analysis, an association model was built taking heart diseases into the consideration. In the group of patients who received 1-2L, heart diseases had enough effect on the regression coefficient to be included into the model, but had no changing effect on association of our concern.  Abbreviations OR= odds ratios, CI= confidence interval, SBP= initial systolic blood pressure, L = liter, PI = Predisposition, Infection, MICU= medium care or intensive care | | |

| **Appendix B3.** Association model for >2 L | | |
| --- | --- | --- |
| Variables included in the model | **OR (95% CI)** | **p-value** |
| **SBP ≤ 120 mmHg** |  |  |
| Age category | 1.71 (0.73 – 3.99) | 0.222 |
| DNR-status | 3.94 (1.54 – 10.07) | 0.004 |
| MICU admission | 5.82 (2.35 – 14.60) | 0.000 |
| **SBP > 120 mmHg** |  |  |
| Age category | 1.99 (0.55 – 7.15) | 0.293 |
| DNR-status | 2.27 (0.62 – 8.32) | 0.215 |
| MICU admission | 2.60 (0.71 – 9.49) | 0.147 |
| For a sensitivity analysis, an association model was built taking heart diseases into the consideration. In the group of patients who received >2L, heart diseases had too little effect on the regression coefficient to be included into the model and so did not affect the association of concern. Abbreviations OR= odds ratios, CI= confidence interval, SBP= initial systolic blood pressure, L = liter, MICU= medium care or intensive care | | |
